# Respiratory syncytial virus hospitalisations among young children: a data linkage study

**DOI:** 10.1017/S0950268819001377

**Published:** 2019-07-29

**Authors:** Namrata Prasad, E. Claire Newbern, Adrian A. Trenholme, Tim Wood, Mark G. Thompson, Nayyereh Aminisani, Q. Sue Huang, Cameron C. Grant

**Affiliations:** 1 Institute of Environmental Science and Research, Wallaceville, New Zealand; 2 Department of Paediatrics: Child & Youth Health, University of Auckland, Auckland, New Zealand; 3 Counties Manukau District Health Board, Auckland, New Zealand; 4 Influenza Division, Centers for Disease Control and Prevention, Atlanta, GA, USA; 5 Neyshabur University of Medical Sciences, Neyshabur, Iran; 6 General Paediatrics, Starship Children's Hospital, Auckland, New Zealand

**Keywords:** Infectious disease epidemiology, paediatrics, respiratory infections, respiratory syncytial virus

## Abstract

We aimed to provide comprehensive estimates of laboratory-confirmed respiratory syncytial virus (RSV)-associated hospitalisations. Between 2012 and 2015, active surveillance of acute respiratory infection (ARI) hospitalisations during winter seasons was used to estimate the seasonal incidence of laboratory-confirmed RSV hospitalisations in children aged <5 years in Auckland, New Zealand (NZ). Incidence rates were estimated by fine age group, ethnicity and socio-economic status (SES) strata. Additionally, RSV disease estimates determined through active surveillance were compared to rates estimated from hospital discharge codes. There were 5309 ARI hospitalisations among children during the study period, of which 3923 (73.9%) were tested for RSV and 1597 (40.7%) were RSV-positive. The seasonal incidence of RSV-associated ARI hospitalisations, once corrected for non-testing, was 6.1 (95% confidence intervals 5.8–6.4) per 1000 children <5 years old. The highest incidence was among children aged <3 months. Being of indigenous Māori or Pacific ethnicity or living in a neighbourhood with low SES independently increased the risk of an RSV-associated hospitalisation. RSV hospital discharge codes had a sensitivity of 71% for identifying laboratory-confirmed RSV cases. RSV infection is a leading cause of hospitalisation among children in NZ, with significant disparities by ethnicity and SES. Our findings highlight the need for effective RSV vaccines and therapies.

## Introduction

Respiratory syncytial virus (RSV) is a common aetiological agent in acute respiratory infections (ARI) [1]; however, uncertainties in RSV burden estimates among children remain. Reported RSV incidence rates vary, with annual hospitalisation rates for RSV-associated ARI among children aged <1 year in high-income countries ranging from 17/1000 to 40/1000 per year [2].

The methods used to quantify RSV disease burden have also varied considerably. Some studies are based on hospital discharge records [3, 4], which rely on passive surveillance for case ascertainment and may lack laboratory confirmation, while others have used indirect statistical methods to quantify RSV attributable burdens [5, 6]. Finally, RSV laboratory methods have evolved, with real-time polymerase chain reaction (PCR) having a higher sensitivity than previously used immunofluorescence and virus isolation techniques [7]. It is unclear whether varied estimates of RSV disease burden are due to hospital coding, testing, other methodological differences or reflect true geographic and seasonal variation; regardless, they highlight the value of active surveillance of RSV with real-time PCR laboratory confirmation.

Only one RSV-specific immunoprophylaxis agent is currently available, but is not considered cost-effective in New Zealand (NZ) [8]. Several RSV vaccine and immunoprophylaxis candidates are currently in development [9] with the most advanced maternal RSV vaccine candidate likely to be available for clinical use within the next 5 years. Comprehensive, country-specific RSV burden estimates are essential to guide the introduction of such interventions.

This study linked active, population-based surveillance and individual-level administrative datasets to estimate the incidence of laboratory-confirmed RSV-associated hospitalisations and their direct healthcare costs among children aged <5 years in Auckland, NZ. Additionally, we compared RSV disease estimates determined through active surveillance to those derived from hospital discharge codes.

## Methods

In this study, we retrospectively followed a cohort of children aged <5 years residing in central, southern and eastern Auckland in 2012–2015 for ARI- and RSV-confirmed hospitalisations. This was done using linked administrative datasets and active ARI hospital surveillance as part of the Southern Hemisphere Influenza Vaccine Effectiveness and Research (SHIVERS) project [10]. The SHIVERS study areas are predominantly urban and based on Statistics NZ population estimates included approximately 71 770 children aged <5 years in 2015, of whom 14% were Māori (NZ's indigenous population), 22% Asian, 27% Pacific (including ethnic groups from Samoa, Cook Islands, Tonga, Niue, Fiji, Tokelau, Tuvalu and Kiribati) and 35% were of European or other ethnicities [11]. Ethical approval for the SHIVERS project was obtained from the NZ Health and Disabilities Ethics Committee (NTX/11/11/102).

### Hospital surveillance

The SHIVERS project was an active, ARI surveillance project conducted in two public hospitals serving the central, southern and eastern region of Auckland that provide all inpatient services for the population residing in the area. From 30 April 2012 to 31 December 2015, research nurses reviewed daily records to identify all overnight admissions with suspected ARI. All patients meeting the WHO severe acute respiratory infection (SARI) case definition, defined as cough and measured or reported fever within the last 7 days (in 2012) and 10 days (from 2013 onwards) were enrolled [12]. Study nurses obtained consent and collected nasopharyngeal swabs from the cases.

To provide an understanding of the respiratory virus hospitalisation burden among ARI patients not meeting the SARI definition, between 2013 and 2015, study nurses enrolled samples of non-SARI respiratory patients, i.e. patients with cough and/or measured or reported fever but not both within 10 days. Sampling in 2013 was during the peak winter period (12 August to 6 October) and included a weekly random selection of two paediatric inpatients who fit the non-SARI respiratory definition at all participating facilities. In 2014 and 2015, this surveillance was extended to randomly enrol approximately six paediatric non-SARI respiratory patients weekly in both hospitals from week 18 to 39 (end of April to end of September).

In addition to respiratory virus test results generated by SHIVERS surveillance, hospital laboratories also provided results from clinical-ordered tests performed on SARI and non-SARI respiratory patients during the study period. These results were included after validation of the hospital assay performance [13].

**Fig. 1.**
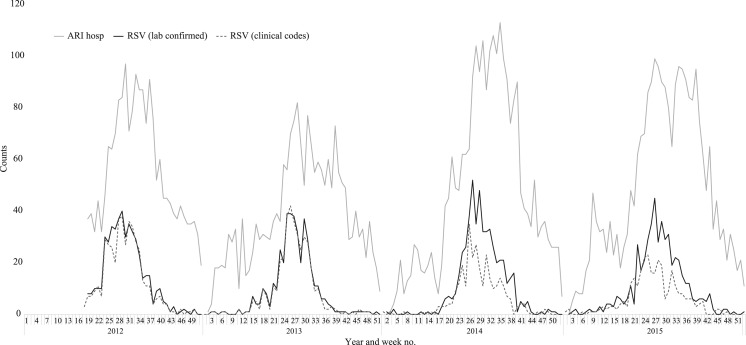
Weekly counts of acute respiratory infection (ARI) hospitalisations, RSV laboratory-confirmed hospitalisations and RSV ICD-10 coded hospitalisations in Auckland, NZ, 2012–2015. RSV laboratory-confirmed cases include all SARI and non-SARI samples tested via SHIVERS study protocol as well as any samples tested for clinical purposes.

Year-round surveillance data showed that RSV followed a distinct seasonal pattern, with 91.8% of RSV-confirmed cases detected during this winter season in NZ (Fig. 1). Since SHIVERS non-SARI respiratory case testing was only conducted during winter, the current analysis was limited to the defined winter season (week 18–39) of 2012–2015.

### Laboratory methods

Specimens were tested for RSV, influenza, rhinovirus (RV), adenovirus (ADV), and human metapneumovirus (hMPV) using the United States Centers for Disease Control and Prevention real-time reverse-transcription (RT)-PCR protocol [14, 15] at the Institute of Environmental Science and Research or the AusDiagnostic PCR protocol and real-time RT-PCR assay at hospital laboratories [16]. A sample of positive specimens was further subtyped.

### Incidence rate denominator

We used two national administrative datasets managed by the NZ Ministry of Health to retrospectively identify children <5 years old residing in the SHIVERS study area during our surveillance period. Individuals in these datasets had a unique National Health Index (NHI) number, which was used for dataset linking.

#### Primary Health Organisation Enrolment Collection

The Primary Health Organisation (PHO) dataset contains demographic data (age, sex, ethnicity, socio-economic status (SES)) for patients enrolled with primary health care facilities [17]. Children who had residential mesh-block data from study areas and were enrolled in primary health care facilities for at least 3 months during a year were classified as residents for that year.

#### National Minimum Dataset

The National Minimum Dataset (NMDS) contains demographic and clinical data on hospitalisations throughout NZ, including all birth events [18]. The NMDS enabled capture of children who were study area residents, not registered with a GP, but had a hospital presentation.

The PHO dataset identified approximately 98% of our study population with the NMDS identifying an additional 2%. Each child was followed from birth or their first year of residence in Auckland until the end of the study period, 5 years of age, movement out of Auckland or death. Each child's time at risk during our defined winter season was calculated. A child could contribute time at risk in multiple age groups (<3, 3–5, 6–11, 12–23 months, 2–4 years) depending on their birth date, residence and timing of winter seasons. These population data were linked to the SHIVERS dataset using NHI numbers to identify those with ARI- and RSV-associated hospitalisations.

### Comparison to hospital discharge codes

**Fig. 2.**
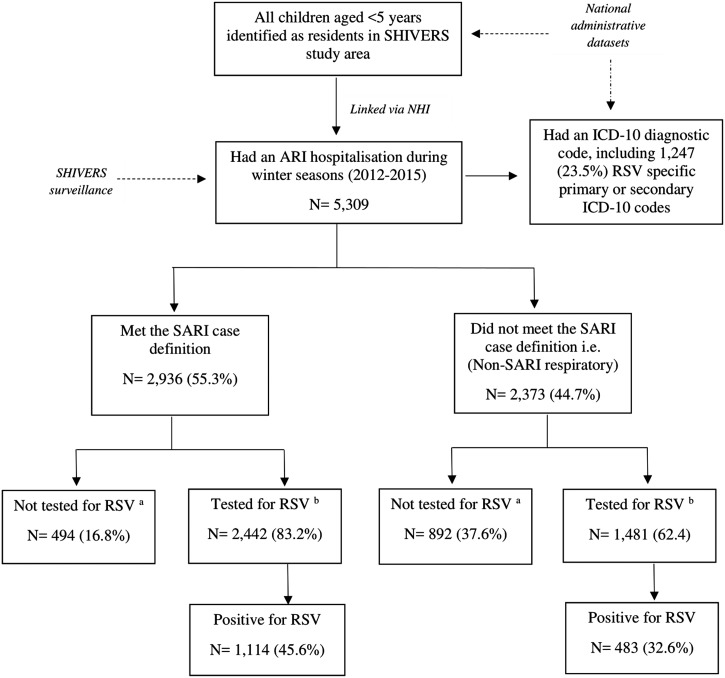
Flowchart detailing retrospective cohort of children aged <5 years in Auckland, New Zealand in 2012–2015 and number of RSV laboratory confirmed and/or an RSV hospital admissions identified by International Classification of Diseases (ICD), 10th edition (ICD-10) diagnostic codes. ^a^For incidence rate calculations, correction of non-testing among ARI patients was done using multivariate imputation by chained equations (MICE) method of imputation (MICE Stata). ^b^Includes both SHIVERS systematic testing results and any results from samples tested for clinical purposes.

All ARI hospitalisations also had an International Classification of Diseases (ICD), 10th edition (ICD-10) diagnostic code, enabling us to compare hospitalisation incidence obtained through active surveillance with laboratory confirmation to incidence based on hospital discharge codes. We classified a child as having an RSV event based on hospital discharge codes if they had at least one primary or secondary RSV-specific ICD-10 code (Table 1). A flowchart illustrating the different components of the study is provided in Figure 2.

**Table 1.** International classification of diseases, 10th edition (ICD-10) diagnostic codes used to identify respiratory syncytial virus (RSV)-associated hospitalisations among children aged <5 years in Auckland, NZ, 2012–2015

Diagnostic category	ICD-10 code^a^	Number identified in the study	Percentage of all acute respiratory hospitalisations *N* = 5309
Primary ICD-10 codes			
RSV as the cause of disease classified to other chapters	B974	88	(1.7)
RSV pneumonia	J121	235	(4.4)
Acute bronchiolitis due to respiratory syncytial virus	J210	862	(16.2)
Acute bronchitis due to respiratory syncytial virus	J205	4	(0.1)
Secondary ICD-10 codes only			
RSV pneumonia	J121	22	(0.4)
Acute bronchiolitis due to respiratory syncytial virus	J210	36	(0.7)
Acute bronchitis due to respiratory syncytial virus	J205	0	–
Total	–	1247	(23.5)

a International Classification of Diseases (ICD), 10th edition (ICD-10) diagnostic code for hospital inpatient cases are reported in the National Minimum Dataset.

### Cost estimation

Each inpatient event and related clinical codes in the NMDS is also allocated a Diagnosis Related Group (DRG) code. The DRG coding system categorises hospitalisations into clinically similar events with comparable resource use. These codes, together with information on hospital length of stay and additional interventions such as mechanical ventilation are used to calculate a cost weight and therefore a cost for each inpatient hospital admission [19]. We calculated hospitalisation costs by multiplying each cost weight with the NZ fixed cost multiplier applicable for the 2017/18 financial year.

### Statistical analysis

For incidence calculations, if a child with confirmed RSV was transferred to another hospital or had two or more RSV-positive hospitalisations within 14 days, they were considered a singular RSV event. We calculated seasonal incidence rates in two ways: (1) calculating the number of singular RSV-associated ARI hospitalisations (events) divided by the number of children residing in the study area during a season; and (2) dividing the number of events by time at risk during each winter season measured as child-years. The first definition was used to enable comparison to other RSV hospitalisation rates, while the second definition was used to have a more precise denominator of time at risk during the surveillance period.

Incidence was stratified by age, ethnicity and SES as they are considered key modifiers of RSV infection risk [1, 20, 21]. SES was quantified using a small-area measure of neighbourhood deprivation derived from the national census (NZDep2013) [22] and was used to separate the cohort into SES quintiles. Confidence intervals for incidence and rate ratios were based on the Poisson distribution. Māori and Pacific people are over-represented in lower SES groups in NZ [23], which may confound the relationship of ethnicity and SES with RSV hospitalisation risk; therefore, the rates and rate ratios relating to these exposures were adjusted for each other to evaluate the independence of their effects.

The *χ*^2^ tests were used to test for associations between categorical variables. The sensitivity, specificity and positive predictive value (PPV) of RSV hospital discharge codes for identifying RSV laboratory-confirmed hospitalisations were also calculated. All analyses were performed using Stata 14 (StataCorp LP, Texas, USA).

#### Correction for non-testing for incidence rates

We verified that non-tested patients were missing at random using a Missing Completely at Random Test [24]. Using the multivariate imputation by chained equations (MICE) method of imputation in STATA (MICE Stata), we created 30 imputed datasets of RSV results with age in months, SARI case status, sex, ethnicity, SES, hospitalisation week and specimen type (clinician-order *vs.* SHIVERS systematic sampling) included as predictors of missingness. Non-imputed results are provided in online Supplementary Table S1.

## Results

### Study population

During 2012–2015, an average of 84 950 children aged <5 years resided in the study area annually. In total, the children in our cohort contributed 131 683 child-years at risk during the winter surveillance period (Table 2).

**Table 2.** Seasonal incidence rates of laboratory-confirmed and ICD-10 coded respiratory syncytial virus (RSV)-associated hospitalisations among children aged <5 years, by year, sub-region, age group, sex, socio-economic status (SES) and ethnicity in Auckland, New Zealand, 2012–2015

	Child time at risk (years)	All ARI	ARI tested for RSV	RSV laboratory-confirmed hospitalisations (adjusted for non-testing)						ICD-10 coded RSV hospitalisations				
				No.	(%) of tested	Rate per 1000 children^a^		Rate per 1000 child-years^a^			Rate per 1000 children^a^		Rate per 1000 child-years^a^	
						IR	(95% CI)	IR	(95% CI)	No.	IR	(95% CI)	IR	(95% CI)
Total	131 683	5309	3923	1597	(40.7)	6.1	(5.8–6.4)	15.0	(14.3–15.7)	1187	9.5	(8.9–10.0)	3.7	(3.5–3.9)
Year														
2012	33 258	1315	931	417	(44.8)	6.8	(6.3–7.4)	17.5	(15.8–19.2)	378	11.8	(10.7–13.0)	4.6	(4.2–5.1)
2013	33 066	1128	823	354	(43.0)	5.5	(5.0–6.0)	14.0	(12.6–15.5)	339	10.4	(9.3–11.5)	4.1	(3.6–4.5)
2014	32 784	1436	1111	442	(39.8)	6.4	(5.9–7.0)	16.5	(14.9–18.1)	244	8.0	(7.0–9.0)	3.1	(2.7–3.5)
2015	32 575	1430	1058	384	(36.3)	5.9	(5.4–6.5)	15.3	(13.8–16.2)	226	7.4	(6.4–8.3)	2.9	(2.5–3.3)
Sub-region														
Central	55 552	2176	1296	504	(38.9)	5.6	(5.2–6.0)	14.6	(13.4–15.7)	378	6.8	(6.1–7.5)	2.6	(2.3–2.8)
South, East	76 130	3133	1670	1093	(65.4)	6.5	(6.1–6.8)	16.5	(15.5–17.5)	809	11.3	(10.6–12.1)	4.5	(4.2–4.8)
Age group														
<3 months	6254	1151	924	450	(48.7)	35.1	(32.2–38.1)	90.3	(82.4–98.3)	412	68	(61.4–74.5)	26.5	(24.0–29.1)
3 to <6 months	6428	828	696	314	(45.1)	22.3	(20.1–24.7)	57.4	(51.2–63.6)	255	41.2	(36.1–46.3)	16.0	(14.1–18.0
6 to <12 months	12 961	1213	958	363	(37.9)	13.2	(12.0–14.5)	33.9	(30.3–37.5)	254	20.4	(17.9–22.9)	8.0	(7.1–9.0)
1 to <2 years.	26 068	1042	737	300	(40.7)	6.0	(5.5–6.7)	15.4	(13.7–17.1)	193	7.8	(6.7–8.9)	3.1	(2.7–3.5)
2 to <5 years.	79 972	1075	608	170	(28.0)	1.3	(1.1–1.5)	3.3	(2.8–3.8)	73	1.0	(0.8–1.3)	0.4	(0.3–0.5)
SES^b^,^c^														
1 (least deprived)	16 358	319	184	78	(42.4)	2.9	(2.4–3.4)	7.4	(5.9–9.0)	58	3.6	(2.7–4.5)	1.4	(1.1–1.8)
2	20 097	478	294	133	(45.2)	3.9	(3.4–4.5)	10.2	(8.5–11.9)	100	5.1	(4.1–6.1)	2.0	(1.6–2.4)
3	18 983	499	319	123	(38.6)	3.8	(3.3–4.4)	9.8	(8.2–11.4)	88	5.0	(3.9–6.0)	1.9	(1.6–2.3)
4	18 161	738	520	219	(42.1)	6.3	(5.6–7.0)	16.2	(14.2–18.3)	168	9.6	(8.2–1.1)	3.7	(3.2–4.3)
5 (most deprived)	58 083	3275	2606	1044	(40.1)	8.4	(8.0–8.9)	21.7	(20.3–23.0)	773	14	(13.1–15.0)	5.4	(5.1–5.8)
Ethnicity^b^														
Māori	19 164	1617	1239	509	(41.1)	12.8	(11.8–13.9)	33.1	(30.2–35.9)	384	20.9	(18.8–23.0)	8.1	(7.3–8.9)
Pacific	36 522	2348	1850	720	(38.9)	9.3	(8.7–9.9)	24.0	(22.2–25.8)	539	15.6	(14.3–16.9)	6.0	(5.6–6.7)
Asian	29 179	496	321	141	(43.9)	2.7	(2.3–3.1)	7.0	(5.9–8.0)	94	3.4	(2.7–4.1)	1.3	(1.1–1.6)
European/other	46 818	848	513	227	(44.2)	2.9	(2.6–3.2)	7.5	(6.6–8.4)	170	3.8	(3.2–4.3)	1.5	(1.3–1.7)

a Incidence rates were calculated using two definitions; (1) calculating the number of singular RSV-associated ARI hospitalisations (events) divided by the number of children residing in the study area during a season; and (2) dividing the number of events by time at risk during each surveillance period measured as child-years.

b Rates for SES and ethnicity are unadjusted.

c SES (socio-economic status) quantified into quintiles using a small-area level measure of household deprivation derived from the national census (NZDep2013) [22].

### Hospitalised patients

During the surveillance period, there were 5309 overnight ARI hospitalisations among children aged <5 years, with 433 (8.2%) admitted to ICU and 19 (0.4%) dying in the hospital or within 30 days of admission. The median (interquartile range (IQR)) length of hospital stay for children hospitalised with ARI was 2 (1–3) days.

Prospective or clinical RSV testing was performed on 3923 (73.9%) ARI hospitalisations, including 1597/3923 (40.7%, 95% confidence interval (CI) 39.2–42.2) RSV-positive cases. Among the 729 subtyped RSV infections, 384 (52.7%) were RSV subtype A, 343 (47.1%) were RSV subtype B, and two cases were concurrently infected with RSV subtype A and B. Seasonality of RSV by subtype is presented in online Supplementary Figure S1.

The proportion of ARI hospitalisations requiring ICU admission did not differ significantly by RSV positivity (RSV-positive = 10.1% *vs.* RSV-negative = 10.2%, *P*-value = 0.926). Among RSV-positive children, one child died during hospitalisation and two children died within 30 days of admission. The median (interquartile (IQ)) hospital length of stay of RSV-positive children was 3 (2–4) days.

Of the 1597 RSV-positive cases, 1187 (74.3%) were also tested for other respiratory viruses. Among these RSV-positive samples, 368 (31.0%) were co-infected with other viruses, which included 136 (37.0%) RV co-infections, 114 (31.0%) ADV co-infections, 41 (11.1) influenza co-infections, 14 (3.8%) hMPV co-infections and 63 (17.1%) samples positive for three or more viruses. Overall we found RSV co-infections to be more common among older children than single RSV infections (Table S2). No significant differences in ICU admission or hospital length of stay were found between RSV co-infections and single RSV infections (Table S2).

The seasonal incidence of RSV-associated ARI hospitalisation without accounting for non-tested children was 3.5 (95% CI 3.3–3.7) per 1000 children or 12.2 (95% CI 11.6–12.9) per 1000 child-years at risk (Table S1). Following imputation for non-testing, the incidence of RSV-confirmed hospitalisation among children aged <5 years was 6.1 (95% CI 5.8–6.4) per 1000 children or 15.0 (95% CI 14.3–15.7) per 1000 child-years at risk (Table 2).

**Fig. 3.**
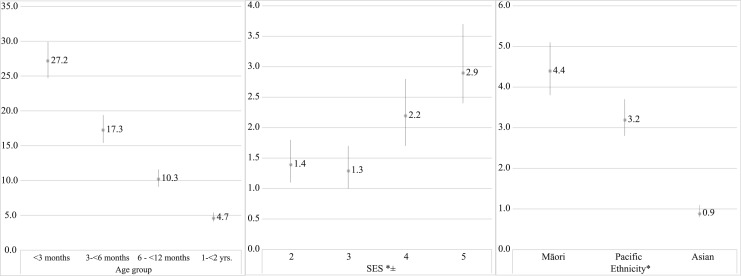
Incidence rate ratios for age group (referent 2 to <5 years old), socio-economic status (referent – quintile 1) and ethnicity (referent – European/other) of RSV-associated ARI hospitalisations among children <5 years of age in Auckland, New Zealand, 2012–2015. *Rate ratios for SES and ethnicity presented in the figure are unadjusted. Adjusted rate ratios are provided in the text. ^±^SES (socio-economic status) quantified into quintiles using a small-area level measure of household deprivation derived from the national census (NZDep2013) [24].

Incidence decreased with age, with children <3 months old being almost 30 times as likely to have an RSV hospitalisation compared to children aged 2 to <5 years (Fig. 3). Māori and Pacific children were at an increased risk of RSV hospitalisation compared with children of European or other ethnicities. Similarly, children from more deprived SES areas were at an increased risk of RSV hospitalisation compared with children from the least deprived areas (Fig. 3). Following adjustment for SES and ethnicity, rates of RSV hospitalisation remained significantly higher in Māori and Pacific children compared with children of European or other ethnicities (Māori rate ratio (RR) 4.0, 95% CI 3.4–4.7; Pacific RR 2.9, 95% CI 2.5–3.4). Children from more deprived SES areas remained at an increased risk compared with children from the least deprived area (lowest SES area RR 1.3, 95% CI 1.0–1.6; second lowest SES area RR 1.4, 95% CI 1.1–1.7).

### Seasonal incidence obtained through hospital discharge codes

During the surveillance periods, 1247 RSV ARI hospitalisations were identified by RSV-specified primary or secondary ICD-10 discharge codes (Table 1). Of RSV-specified codes, 1187 (95.2%) were primary discharge codes (Table 3).

**Table 3.** Laboratory-confirmed RSV-associated hospitalisations and corresponding primary ICD-10 code

Corresponding primary hospital discharge code^a^	ICD-10 code	RSV-positive		RSV-negative		RSV untested		Total	
		*N*	(%)	*N*	(%)	*N*	(%)	*N*	(%)
Total		1597	(100.0)	2251	(100.0)	1347	(100.0)	5195	(100.0)
All RSV-specified		1081	(67.7)	26	(1.2)	80	(5.9)	1187	(22.8)
RSV as the cause of disease classified to other chapters	B974	76	(4.8)	7	(0.3)	4	(0.3)	87	(1.7)
RSV pneumonia	J121	210	(13.1)	5	(0.2)	21	(1.6)	236	(4.5)
Acute bronchiolitis due to respiratory syncytial virus	J210	791	(49.5)	14	(0.6)	55	(4.1)	860	(16.6)
Acute bronchitis due to respiratory syncytial virus	J205	4	(0.3)	0	(0.0)	0	(0.0)	4	(0.1)
Non-RSV specified respiratory		473	(29.6)	1923	(85.4)	1095	(81.3)	3491	(67.2)
Acute upper respiratory infections	J00-J06	11	(0.7)	187	(8.3)	102	(7.6)	300	(5.8)
Acute lower respiratory infections	A37, J09–J20	420	(26.3)	1528	(67.9)	678	(50.3)	2626	(50.5)
Whooping cough	A37	1	(0.1)	24	(1.1)	8	(0.6)	33	(0.6)
Influenza and pneumonia	J09–J18	196	(12.3)	619	(27.5)	257	(19.1)	1072	(20.6)
Bronchiolitis	J21	190	(11.9)	779	(34.6)	365	(27.1)	1334	(25.7)
Unspecified ALRI	J22	32	(2.0)	98	(4.4)	47	(3.5)	177	(3.4)
Bronchitis	J20	1	(0.1)	8	(0.4)	1	(0.1)	10	(0.2)
Other and unspecified asthma	J459	8	(0.5)	84	(3.7)	161	(12.0)	253	(4.9)
Wheezing	R062	34	(2.1)	124	(5.5)	154	(11.4)	312	(6.0)
Non-RSV-specified non-respiratory		43	(2.7)	302	(13.4)	171	(12.7)	516	(9.9)
Viral infection unspecified	B349	4	(0.3)	37	(1.6)	17	(1.3)	58	(1.1)
Other	Xxx	39	(2.4)	265	(11.8)	154	(11.4)	458	(8.8)

a Table 3 is only displaying primary ICD-10 discharge codes, of the 1597 RSV-positive children, 49 (3.1%) had a secondary RSV-specified ICD-10 code.

The seasonal incidence of RSV ICD-10 coded hospitalisations per 1000 children was 3.7 (95% CI 3.5–3.9) or 9.5 (95% CI 8.9–10.0) per 1000 child-years (Table 2). Rates obtained using active, laboratory-confirmed surveillance were 1.7 (95% CI 1.5–1.8) times higher than rates obtained using hospital discharge codes. Active, laboratory-confirmed surveillance rates were twice the rate obtained from hospital discharge codes in 2014 and 2015 (2014 RR 2.1, 95% CI 1.8–2.4; 2015 RR 2.1, 95% CI 1.8–2.4), twice the rate among 1-year-old children (RR 2.0, 95% CI 1.7–2.3), and more than three times the rate in children aged 2–4 years (RR 3.2, 95% CI 2.5–4.2).

Of the 1597 RSV laboratory-confirmed hospitalisations, 1130 (70.8%) had an RSV-specified primary or secondary hospital discharge diagnosis. Compared to RSV PCR positivity, the sensitivity, specificity and PPV of RSV-associated primary or secondary J121, J210, J205 or B974 discharge codes were 71%, 99% and 98%, respectively. Further comparisons of RSV laboratory-confirmed cases to hospital discharge codes are provided in the online Supplementary material (Figures S2 and S3).

### Direct health-care cost

Based on the DRG costing methodology, the median (IQR) cost per RSV hospitalisation was $NZ 3154.96 ($3070.80–$4931.99) or an average of $NZ 5040.20 per episode (Table S3). After accounting for non-testing, the annual estimated direct healthcare cost of RSV-confirmed hospitalisations among children aged <5 years in the Auckland region was $NZ 2.6 million.

### Extrapolation to national population

If our ethnicity-specific RSV hospitalisation rates were extrapolated to the entire NZ population, we estimate approximately 1900 children aged <5 years would have an RSV-associated hospitalisation each year with a direct health care cost of $NZ 8.2 million. Moreover, we estimate Māori and Pacific to represent approximately 41% and 16% of all RSV hospitalisations, despite comprising 20% and 10% of the NZ child population, respectively.

## Discussion

We found that RSV has a high health and economic burden in NZ, accounting for roughly 40% of ARI hospitalisations. Being of Indigenous Māori or Pacific ethnicity or living in more socio-economically deprived areas were independent risk factors for RSV hospitalisation. Relying on RSV hospital discharge codes under-estimated RSV burden, given their sensitivity of 71% for identifying laboratory-confirmed RSV cases.

Our results are similar to a year-round prospective study of RSV in South Auckland among children aged <2 years [25], which suggests that our seasonal incidence approximates to annual RSV incidence. Among NZ children aged <5 years, RSV-associated hospitalisation rates were roughly three times higher than those reported for influenza (IR = 1.9 per 1000 children) during the same period [26]. Rates in our study are also higher than the reported rotavirus hospitalisation rates for NZ children aged <5 years before rotavirus vaccine introduction [27, 28], and highlight the relative contribution of RSV to hospital disease burden. Moreover, we found that an RSV hospitalisation costs approximately $NZ 5040 per episode. This is higher than the average direct cost of rotavirus hospitalisations, which was estimated at $NZ 1512 per episode among children <5 years in 2006/2007 [29] or approximately $NZ 2400 in 2017/18.

When comparing our findings to a US study that used active laboratory-confirmed surveillance, we found our proportion positivity for RSV (40%) to be approximately twice the proportion positive found in the USA, moreover our hospitalisation rate of 6.1 per 1000 children was twice their rate [30]. Our findings are consistent with the national comparative data showing children <2 years old in NZ to be twice as likely to be hospitalised with bronchiolitis or pneumonia as those living in England or the USA [31–35]. Factors such as differences in health care-seeking behaviour and thresholds for hospital admission are likely to be contributing to this disparity. Additionally, the disproportionate burden of RSV disease experienced by Māori and Pacific children may also be contributing to the high burden observed in NZ. While the RSV hospitalisation rates among Māori and Pacific children in our study are comparable to the high rates observed among indigenous populations in Australia [3] and North America [21, 36], in Auckland, Māori and Pacific children comprise approximately 40% of the child population which is in contrast to Australia and North America, where indigenous populations comprise 2–3% of the total population in major urban areas.

Children in the most deprived SES groups also had an increased risk of RSV-associated hospitalisations. Previous studies from NZ have highlighted the disparity in respiratory and other infectious diseases burden [37], linking these to SES and ethnicity-related factors such as household crowding [38], maternal smoking and perceived experience of health care racism [29], as well as barriers to primary health care [39]. Such findings are in-line with our result of independent SES and ethnicity-related effects. The introduction of pneumococcal conjugate and meningococcal group B vaccines has both been associated with reductions in social and ethnic disparities in hospitalisations in NZ [40, 41] and suggests that an RSV vaccine and/or immunoprophylaxis may have a similar impact.

We found laboratory-confirmed hospitalisation rates to be about 1.7 times higher than discharge coded rates overall and more than double discharge coded rates between 2014 and 2015 and among children aged ⩾1 year (Table 2). Protocols at hospitals were reported to change from routine testing of all ARIs to testing based on clinical suspicion between 2014 and 2015 (A Trenholme, personal communication) and may have driven more RSV testing in patients considered at a higher risk. Such findings are useful for other countries relying on passive surveillance and hospital discharge codes to estimate RSV disease burden.

Our study had limitations. First, our population estimates of children aged <5 years are higher than population estimates from Statistics NZ; additionally, we are unable to guarantee that all children classified as residents in our study sought health care at our surveillance sites. However, as the data sources are continually updated and validated and our surveillance sites are the only hospitals providing acute inpatient paediatric care, we considered this approach as the most robust method in estimating hospitalisation rates. Second, our cost estimations did not account for indirect costs associated with the loss of work and out-of-pocket expenses. Finally, we did not have population-level data on well-established risk factors for severe RSV disease such as premature birth, exposure to second-hand smoking and other underlying conditions, preventing the estimation of RSV hospitalisation rates within these strata. Such rates will be valuable in informing RSV vaccine/therapy use among high-risk groups. Nonetheless, the major strength of this study is its use of active laboratory-confirmed surveillance linked with individual-level population data, enabling the estimation of RSV hospitalisation rates by key demographic strata.

## Conclusion

We confirm that RSV is a leading cause of hospitalisation among young children and has a high economic cost. RSV hospitalisation rates in our study are almost twice the rate reported in a similar study from the USA. In NZ, being of Māori or Pacific ethnicity or living in a low socio-economic neighbourhood independently increased the risk of having an RSV-associated hospitalisation. RSV hospitalisation rates obtained though active RSV surveillance are almost twice as high as the rates obtained from hospital discharge code data. Our findings highlight the need for effective RSV vaccines and therapies.

## Supplementary material

For supplementary material accompanying this paper visit https://doi.org/10.1017/S0950268819001377. click here to view supplementary material
