# Respiratory syncytial virus hospitalisations among young children: a data linkage study – Erratum

**DOI:** 10.1017/S0950268820001612

**Published:** 2020-08-28

**Authors:** Namrata Prasad, E. Claire Newbern, Adrian A. Trenholme, Tim Wood, Mark G. Thompson, Nayyereh Aminisani, Q. Sue Huang, Cameron C. Grant

**Affiliations:** 1 Institute of Environmental Science and Research, Wallaceville, New Zealand; 2 Department of Paediatrics: Child &Youth Health, University of Auckland, Auckland, New Zealand; 3 Counties Manukau District Health Board, Auckland, New Zealand; 4 Influenza Division, Centers for Disease Control and Prevention, Atlanta, GA, USA; 5 NeyshaburUniversity of Medical Sciences, Neyshabur, Iran; 6 General Paediatrics, Starship Children's Hospital, Auckland, New Zealand

The original version of this article was published with an error in Table 2. The data in the last two columns (Rate per 1000 children and Rate per 1000 child-years) was transposed and displayed under the incorrect heading. Please find the corrected version of the table below.

**Table 2.** Seasonal incidence rates of laboratory-confirmed and ICD-10 coded respiratory syncytial virus (RSV) associated hospitalisations among children aged less than 5 years, by year, sub-region, age group, sex, socio-economic status (SES) and ethnicity in Auckland, New Zealand, 2012–2015

				RSV laboratory-confirmed hospitalisations (adjusted for non-testing)						ICD-10 coded RSV hospitalisations				
						Rate per 1000 children^a^		Rate per 1000 child-years^a^			Rate per 1000 children^a^		Rate per 1000 child-years^a^	
	Child time at risk (years)	All ARI	ARI tested for RSV	No.	(%) of tested	IR	(95% CI)	IR	(95% CI)	No.	IR	(95% CI)	IR	(95% CI)
**Total**	131 683	5309	3923	1597	(40.7)	6.1	(5.8–6.4)	15.0	(14.3–15.7)	1247	3.7	(3.5–3.9)	9.5	(8.9–10.0)
Year														
2012	33 258	1315	931	417	(44.8)	6.8	(6.3–7.4)	17.5	(15.8–19.2)	399	4.6	(4.2–5.1)	11.8	(10.7–13.0)
2013	33 066	1128	823	354	(43.0)	5.5	(5.0–6.0)	14.0	(12.6–15.5)	346	4.1	(3.6–4.5)	10.4	(9.3–11.5)
2014	32 784	1436	1111	442	(39.8)	6.4	(5.9–7.0)	16.5	(14.9–18.1)	261	3.1	(2.7–3.5)	8.0	(7.0–9.0)
2015	32 575	1430	1058	384	(36.3)	5.9	(5.4–6.5)	15.3	(13.8–16.2)	241	2.9	(2.5–3.3)	7.4	(6.4–8.3)
Sub-region														
Central Auckland	55 552	2176	1296	504	(38.9)	5.6	(5.2–6.0)	14.6	(13.4–15.7)	374	2.6	(2.3–2.8)	6.8	(6.1–7.5)
South East Auckland	76 130	3133	1670	1093	(65.4)	6.5	(6.1–6.8)	16.5	(15.5–17.5)	873	4.5	(4.2–4.8)	11.3	(10.6–12.1)
Age group														
0–2 months	6254	1151	924	450	(48.7)	35.1	(32.2–38.1)	90.3	(82.4–98.3)	427	26.5	(24.0–29.1)	68	(61.4–74.5)
3–5 months	6428	828	696	314	(45.1)	22.3	(20.1–24.7)	57.4	(51.2–63.6)	265	16.0	(14.1–18.0	41.2	(36.1–46.3)
6–11 months	12 961	1213	958	363	(37.9)	13.2	(12.0–14.5)	33.9	(30.3–37.5)	267	8.0	(7.1–9.0)	20.4	(17.9–22.9)
12–23 months.	26 068	1042	737	300	(40.7)	6.0	(5.5–6.7)	15.4	(13.7–17.1)	206	3.1	(2.7–3.5)	7.8	(6.7–8.9)
2–4 years.	79 972	1075	608	170	(28.0)	1.3	(1.1–1.5)	3.3	(2.8–3.8)	82	0.4	(0.3–0.5)	1.0	(0.8–1.3)
SES^b^^,^^c^														
1 (least deprived)	16 358	319	184	78	(42.4)	2.9	(2.4–3.4)	7.4	(5.9–9.0)	59	1.4	(1.1–1.8)	3.6	(2.7–4.5)
2	20 097	478	294	133	(45.2)	3.9	(3.4–4.5)	10.2	(8.5–11.9)	103	2.0	(1.6–2.4)	5.1	(4.1–6.1)
3	18 983	499	319	123	(38.6)	3.8	(3.3–4.4)	9.8	(8.2–11.4)	94	1.9	(1.6–2.3)	5.0	(3.9–6.0)
4	18 161	738	520	219	(42.1)	6.3	(5.6–7.0)	16.2	(14.2–18.3)	175	3.7	(3.2–4.3)	9.6	(8.2–1.1)
5 (most deprived)	58 083	3275	2606	1044	(40.1)	8.4	(8.0–8.9)	21.7	(20.3–23.0)	816	5.4	(5.1–5.8)	14.0	(13.1–15.0)
Ethnicity^b^														
Māori	19 164	1617	1239	509	(41.1)	12.8	(11.8–13.9)	33.1	(30.2–35.9)	401	8.1	(7.3–8.9)	20.9	(18.8–23.0)
PI	36 522	2348	1850	720	(38.9)	9.3	(8.7–9.9)	24.0	(22.2–25.8)	570	6.0	(5.6–6.7)	15.6	(14.3–16.9)
Asian	29 179	496	321	141	(43.9)	2.7	(2.3–3.1)	7.0	(5.9–8.0)	100	1.3	(1.1–1.6)	3.4	(2.7–4.1)
European/other	46 818	848	513	227	(44.2)	2.9	(2.6–3.2)	7.5	(6.6–8.4)	176	1.5	(1.3–1.7)	3.8	(3.2–4.3)

a Incidence rates were calculated using two definitions; (1) calculating the number of singular RSV-associated ARI hospitalisations (events) divided by the number of children residing in the study area during a season; and (2) dividing the number of events by time at risk during each surveillance period measured as child-years.

b Rate for SES and ethnicity presented in the table are unadjusted. Adjusted rate ratios are provided in the text.

c SES (socioeconomic status) quantified into quintiles using a small area-level measure of household deprivation derived from the national census (NZDep2013).
