# Supplementary material for: Respiratory syncytial virus hospitalisations among young children: a data linkage study
Source: Epidemiol Infect. 2019 Jul 29;147:e246. doi: 10.1017/S0950268819001377 (PMC6805750; doi:10.1017/S0950268819001377)
Supplement: Supplementary file 1 [file S0950268819001377sup001.docx]

# Supplementary material

# Table S1: Seasonal incidence rates of all acute respiratory (ARI), lab confirmed and ICD-10 coded respiratory syncytial virus (RSV) associated hospitalizations among children aged <5 years, by socio-economic status (SES), and ethnicity in Auckland, New Zealand, 2012-2015 *not corrected for non-testing*

|  | RSV lab confirmed hospitalization rates (NOT corrected for non-testing) | | | | | | | | | |
| --- | --- | --- | --- | --- | --- | --- | --- | --- | --- | --- |
|  | No. | Rate per 1000 child years at risk | | | | Rate per 1000 children | | | | |
|  |  | IR | (95% CI) | RR | (95% CI) | IR | | (95% CI) | RR | (95% CI) |
| Total | 1597 | 12.2 | (11.62-12.86) |  |  | 4.7 | (4.5-5.0) | |  |  |
| Year |  |  |  |  |  |  |  | |  |  |
| 2012 | 417 | 12.5 | (11.3-13.7) |  |  | 4.8 | (4.4-5.3) | |  |  |
| 2013 | 354 | 10.7 | (9.5-11.8) |  |  | 4.1 | (3.7-4.6) | |  |  |
| 2014 | 442 | 13.5 | (12.2-14.8) |  |  | 5.2 | (4.7-5.7) | |  |  |
| 2015 | 384 | 11.8 | (10.6-13.0) |  |  | 4.6 | (4.1-5.0) | |  |  |
| Sub-region |  |  |  |  |  |  |  | |  |  |
| Auckland | 504 | 9.2 | (8.4-10.0) |  |  | 3.5 | (3.2-3.8) | |  |  |
| Counties Manukau | 1093 | 14.4 | (13.6-15.3) |  |  | 5.6 | (5.3-6.0) | |  |  |
| Age Group |  |  |  |  |  |  |  | |  |  |
| <3 months | 450 | 72.4 | (65.7-79.1) | 33.7 | (30.1-37.6) | 27.9 | (25.3-30.5) | |  |  |
| 3-<6 months | 314 | 48.9 | (43.4-54.3) | 22.9 | (20.1-26.0) | 18.9 | (16.8-21.0) | |  |  |
| 6 - <12 months | 363 | 27.9 | (24.9-30.9) | 13.1 | (11.4-15.0) | 10.8 | (9.6-11.9) | |  |  |
| 1-<2 year | 300 | 11.4 | (10.1-12.7) | 5.4 | (4.6-6.4) | 4.4 | (3.9-4.9) | |  |  |
| 2-<5 year | 170 | 2.1 | (1.8-2.4) | Ref |  | 0.8 | (0.7-0.9) | |  |  |
| SES*† |  |  |  |  |  |  |  | |  |  |
| 1 (least deprived) | 78 | 8.9 | (6.8-11.0) | Ref |  | 3.5 | (2.6-4.3) | | Ref |  |
| 2 | 133 | 10.4 | (8.5-12.3) | 1.2 | (0.9-1.6) | 4.0 | (3.3-4.7) | | 1.2 | (0.9-1.5) |
| 3 | 123 | 9.2 | (7.6-10.9) | 1.0 | (0.8-1.4) | 3.6 | (2.9-4.2) | | 1.0 | (0.8-1.4) |
| 4 | 219 | 13.0 | (11.2-14.7) | 1.5 | (1.1-1.9) | 5.0 | (4.4-5.7) | | 1.5 | (1.1-1.9) |
| 5 (most deprived) | 1044 | 13.3 | (12.4-14.2) | 1.5 | (1.2-1.9) | 5.2 | (4.8-5.5) | | 1.5 | (1.2-1.9) |
| Ethnicity† |  |  |  |  |  |  |  | |  |  |
| Maori | 509 | 25.0 | (22.7-27.4) | 4.7 | (3.9-5.6) | 9.7 | (8.8-10.6) | | 4.7 | (3.9-5.6) |
| Pacific | 720 | 18.1 | (16.6-19.7) | 3.4 | (2.9-4.0) | 7.0 | (6.4-7.6) | | 3.4 | (2.8-4.0) |
| Asian | 141 | 5.0 | (4.2-5.9) | 0.9 | (0.8-1.2) | 2.0 | (1.6-2.3) | | 0.9 | (0.8-1.2) |
| European/Other | 227 | 5.3 | (4.6-6.1) | Ref |  | 2.1 | (1.8-2.4) | | Ref |  |

*SES (Socioeconomic status) quantified using a small area level measure of neighborhood deprivation derived from the national census (NZDep2013) [27]

† Rates for ethnicity and SES have been adjusted for each other.

Table S2. Comparison of age group and clinical outcomes in single RSV infections vs RSV co-infections among children <5 years in Auckland, New Zealand, 2012-2015

|  | RSV single | | RSV co-infection | | p-value |
| --- | --- | --- | --- | --- | --- |
|  | N | (%) | N | (%) |  |
| Total | 819 | (100.0) | 368 | (100.0) |  |
| Age Grp |  |  |  |  |  |
| <3 months | 197 | (24.1) | 62 | (16.8) | 0.005 |
| 3-<6 months | 142 | (17.3) | 68 | (18.5) | 0.634 |
| 6 - <12 months | 195 | (23.8) | 122 | (33.2) | 0.000 |
| 1-<2 yrs. | 168 | (20.5) | 76 | (20.7) | 0.956 |
| 2-<5 yrs. | 117 | (14.3) | 40 | (10.9) | 0.108 |
| Clinical outcomes |  |  |  |  |  |
| ICU admission | 67 | (8.2) | 30 | (8.2) | 0.986 |
| LOS – median (range) | 3 | (1-12) | 2 | (1-13) | 0.420 |

Table S3. Direct health care associated cost for RSV positive hospitalizations by age groups using Diagnosis Related Group (DRG) cost weights in Auckland, New Zealand, 2012-2015

|  | **No. of episodes** | **Total cost ^a^** | **Median (IQR) cost** | **Average cost per episode** | **Annual cost ($NZD) in study area** | **Annual cost ($USD) in study area ^b^** |
| --- | --- | --- | --- | --- | --- | --- |
| RSV positive ^c^ | 1615 | $8,139,923.05 | $3154.96 ($3070.80-$4931.99) | $5,040.20 | $2,034,980.76 | $1,400,575.51 |
| Imputed estimate ^d^ | 2066 | $10,413,053.27 |  |  | $2,603,263.32 | $1,791,695.98 |
| By age group |  |  |  |  |  |  |
| <3 months | 459 | $2,952,299.00 | $4241.55 ($3070.80-$6192.79) | $6,432.02 | $738,074.75 | $507,979.95 |
| 3-<6 months | 319 | $1,607,981.00 | $3169.23 ($3070.80-$4931.99) | $5,040.69 | $401,995.25 | $276,673.23 |
| 6 - <12 months | 365 | $1,701,770.00 | $3154.96 ($3070.80-$4931.99) | $4,662.38 | $425,442.50 | $292,810.80 |
| 1-<2 | 302 | $1,297,951.00 | $3132.81 ($3039.31-$4318.81) | $4,297.85 | $324,487.75 | $223,328.69 |
| 2-<5 | 170 | $579,921.30 | $3132.81 ($3039.31-$3169.23) | $3,411.30 | $144,980.33 | $99,782.71 |

^a^ Total cost between 2012-2015 is calculated as the sum of each individual hospitalization event cost weight multiplied by the New Zealand fixed cost multiplier for the 2017/18 financial year which was equal to $4921.16.

^b^ Currency conversion was based on currency exchange rates from November, 2018.

^c^ RSV positives include non-unique events i.e. if a child confirmed to have RSV was transferred to another hospital or had two or more RSV positive hospitalizations within 14 days of discharge from their first event. While these events are possibly the same RSV episode they are indicative of health care utilisation and cost and are therefore included in cost estimations.

^d^ The average cost per episode for RSV positive hospitalizations was multiplied to the imputed RSV positive estimate to obtain the total, and annual cost.

Figure S1: Weekly counts of RSV laboratory confirmed hospitalizations by RSV subtype in Auckland, NZ, 2012-2015

Figure S2: Comparison of counts of RSV lab confirmed events to primary ICD-10 clinical codes by week and year in the Auckland and Counties Manukau District Health Boards (ADHB, CMDHB)

Figure S3: Comparison of counts of RSV lab confirmed events to primary ICD-10 clinical codes by week and year among SARI and non-SARI respiratory cases
